# Bio-inspired visual self-localization in real world scenarios using Slow Feature Analysis

**DOI:** 10.1371/journal.pone.0203994

**Published:** 2018-09-21

**Authors:** Benjamin Metka, Mathias Franzius, Ute Bauer-Wersing

**Affiliations:** 1 Faculty of Computer Science and Engineering, Frankfurt University of Applied Sciences, Frankfurt am Main, Hessen, Germany; 2 Honda Research Institute Europe, Offenbach am Main, Hessen, Germany; Naval Surface Warfare Center, UNITED STATES

## Abstract

We present a biologically motivated model for visual self-localization which extracts a spatial representation of the environment directly from high dimensional image data by employing a single unsupervised learning rule. The resulting representation encodes the position of the camera as slowly varying features while being invariant to its orientation resembling place cells in a rodent’s hippocampus. Using an omnidirectional mirror allows to manipulate the image statistics by adding simulated rotational movement for improved orientation invariance. We apply the model in indoor and outdoor experiments and, for the first time, compare its performance against two state of the art visual SLAM methods. Results of the experiments show that the proposed straightforward model enables a precise self-localization with accuracies in the range of 13-33cm demonstrating its competitiveness to the established SLAM methods in the tested scenarios.

## Introduction

Many animals have excellent navigation capabilities and outperform current technical systems especially in terms of robustness. In rodents spatial information is encoded by different cell types in the hippocampus. Place cells and head-direction cells encode the position and orientation of the animal and are strongly driven by visual input [1]. The brain is able to extract such high level information from the raw visual data received by the retina. While the sensory signals of single receptors may change very rapidly, e.g., even by slight changes in orientation, the brain’s high level representations of position and orientation typically change on a much lower timescale. This observation has led to the concept of slowness learning [2–5].

It has already been demonstrated that a hierarchical Slow Feature Analysis (SFA) network applied to the visual input of a virtual rat can model place cells and head-direction cells [6, 7]. Recordings from rats’ place cells in open field experiments typically show that cells encode the animal’s own position while being invariant to head direction. Theoretical analysis of the biomorphic model in [7] has shown that in slowness learning, the resulting representation strongly depends on the movement statistics of the animal. Position encoding with invariance to head direction requires a relatively large amount of head rotation around the yaw axis compared to translational movement during mapping of the environment. While such movement may be realistic for a rodent exploring its environment, it is inefficient for a robot with a fixed camera. An extension to the model, using an uncalibrated omnidirectional imaging system for simulating additional rotational movement, was successfully applied to a mobile robot in an outdoor environment [8].

The ability to perform self-localization is crucial for autonomous mobile robots operating in spatial environments. Research in the last 20 years has investigated methods that enable a robot to perform simultaneous localization and mapping (SLAM) in indoor and outdoor environments using different kinds of sensor modalities like laser, range or image sensors [9, 10]. Vision based localization is especially interesting because of the low cost, weight and high availability of cameras, but is still a field of active research due to the challenges of visual perception in real world environments.

A biologically motivated SLAM approach inspired from rat navigation is RatSLAM [11]. The current pose (*x*, *y*, *θ*) is encoded by an activity packet in a 3d continuous attractor network with axis representing (*x*, *y*, *θ*). Self-motion cues and visual template matching inject energy into the network shifting the peak of activity. The unique combinations of local views and poses are defined as experiences which are organized in a graph like map that enables the model to maintain a consistent spatial representation over extended periods of time. In [12] a 66 kilometer urban road network was successfully mapped with a single webcam. Geometric SLAM approaches mainly use sparse visual features to estimate the ego-motion of the camera and the features’ 3d-position from correspondences between successive frames. Methods fusing ego-motion estimates and sensor readings in a probabilistic framework (e.g. Extended Kalman Filter, Particle Filter) have been proposed [13, 14]. Recent approaches [15–17] represent the map as a pose-graph of keyframes connected by ego motion information and feature observations. Loop closure detections enable the correction of accumulated drift by a global optimization of the pose-graph. The 3d-position of features and camera poses is jointly optimized by local bundle adjustment minimizing the re-projection error. Direct methods do not rely on sparse image features but instead estimate the camera motion and scene depth performing direct image alignment by minimizing the difference in pixel intensities. They make use of the whole image [18], which yields a dense 3d reconstruction, or only image regions with high gradients [19], which requires less computational resources and results in a semi-dense reconstruction of the environment.

Modern SLAM approaches are based on methods which evolved over the last decades. A SLAM system generally consists of a front end that establishes image correspondences and performs ego-motion estimation and loop closure detection. The backend uses the information provided by the front end to build and update the map which involves methods from graph theory, optimization and probabilistic estimation. Its successful application furthermore requires sensor calibration and a careful parameter selection. In comparison, the presented SFA-network is a rather straightforward model for self-localization in the sense that it applies the same unsupervised learning rule in a hierarchical network directly to the images from an uncalibrated image sensor. Furthermore, it has also been shown in [7] that the hierarchical model is robust under a range of parameter settings for image resolution, number of layers, receptive field size and overlap. An advantage of the SLAM methods is that they incrementally build a map of the environment and are able to simultaneously localize within this map. The SFA-model requires an inital offline learning phase, as it is based on a closed form solution for solving a generalized eigenvalue problem, in which the environment is evenly sampled. But once trained, localization is absolute and instantaneous since slow features can be computed from a single snapshot of the environment. Thus, localization is not affected by drift over time and there is no need to deal with re-localization. Besides the even sampling the model has no further restrictions on the movement pattern and is able to deal with pure rotational movement which poses a problem to the aforementioned geometric methods. These properties render the model suitable for service robot scenarios.

We apply the biologically motivated model of SFA localization in small scale open field scenarios in indoor and outdoor environments and compare its performance for the first time with the feature based ORB-SLAM [17] and the semi-dense LSD-SLAM [19]. The methods have been chosen because they allow a metric evaluation, represent the state of the art in monocular visual SLAM and are made available by the authors (https://github.com/raulmur/ORB_SLAM, https://github.com/tum-vision/lsd_slam). Results from the experiments show a competitive localization performance of the straightforward model, based on a single learning rule and uncalibrated hardware.

## Methods

### Slow Feature Analysis

SFA as introduced in [4, 20] solves the following objective: given a multidimensional input signal ***x***(*t*), find instantaneous scalar input-output functions *g*_*j*_(***x***) such that the output signals

$$\begin{array}{c} y_{j}\left(t\right)≔g_{j}\left(x\left(t\right)\right) \end{array}$$ (1)

minimize

$$\begin{array}{c} \Delta (y_{j})≔{\left\langle {\dot{y}}_{j}^{2}\right\rangle }_{t} \end{array}$$ (2)

under the constraints

$$\begin{array}{c} {\left\langle y_{j}\right\rangle }_{t}=0\;\;\;\;(zero\;mean), \end{array}$$ (3)

$$\begin{array}{c} {\left\langle y_{j}^{2}\right\rangle }_{t}=1\;\;\;\;(unit\;variance), \end{array}$$ (4)

$$\begin{array}{c} \forall i<j:{\left\langle y_{i}y_{j}\right\rangle }_{t}=0\;\;\;\;(decorrelation\;and\;order) \end{array}$$ (5)

with 〈⋅〉_*t*_ and $\dot{y}$ indicating temporal averaging and the derivative of *y*, respectively. The Δ-value is a measure of the temporal slowness of the signal *y*_*j*_(*t*). It is given by the mean square of the signal’s temporal derivative, so small Δ-values indicate slowly varying signals. The constraints avoid the trivial constant solution and ensure that different functions *g* code for different aspects of the input. We use the SFA implementation from the Modular toolkit for Data Processing (MDP) [21], which is based on solving a generalized eigenvalue problem.

### Orientation invariance

**Fig 1:**
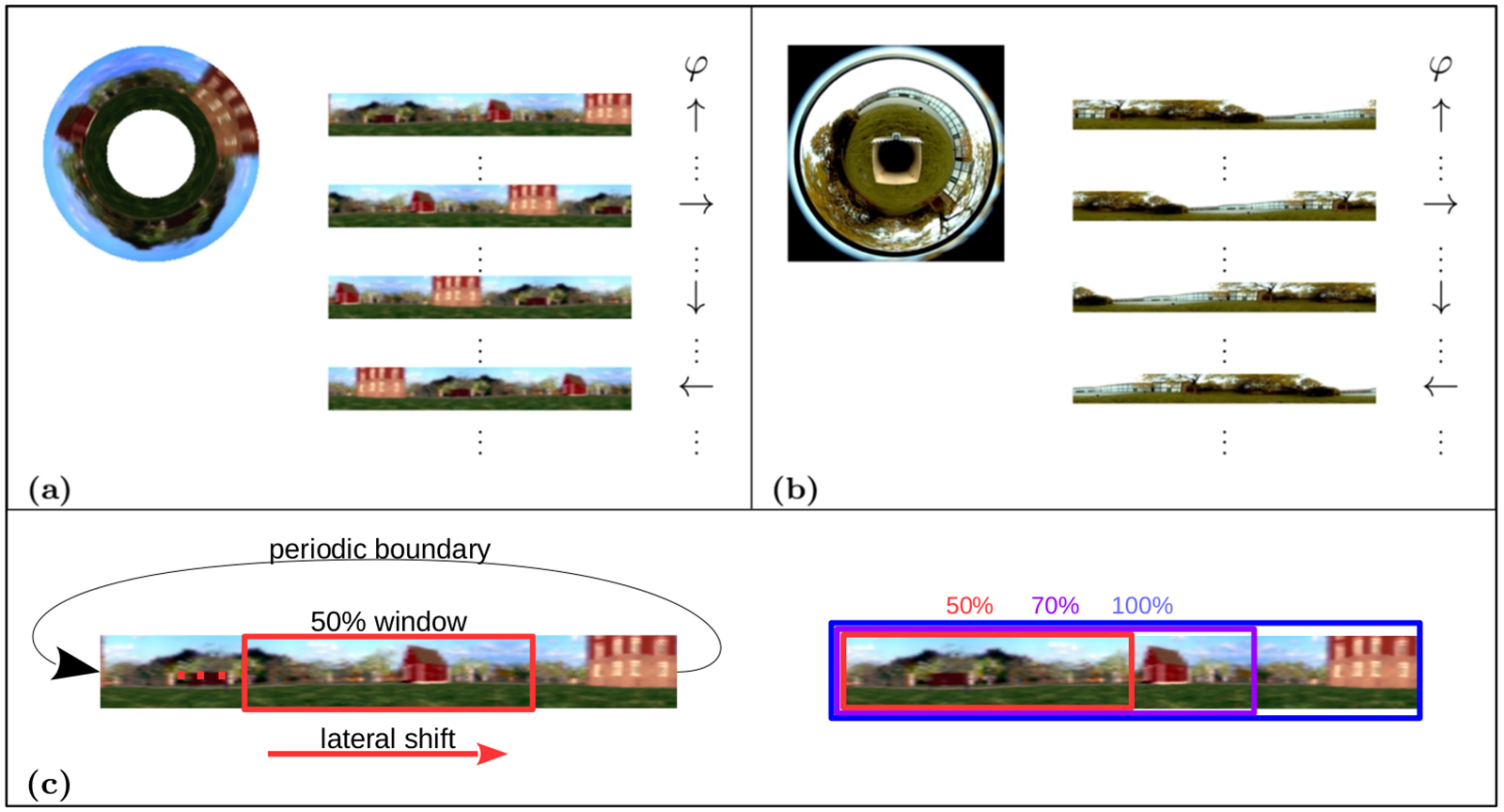
**Simulated rotation for** (a) simulator and (b) real world experiments. The circular image of the surrounding is transformed to a panoramic view with periodic boundaries. Rotation is simulated for every view from one location by laterally sliding a window over the panoramic image with increments of 5 pixels. Thus the variable *φ* denotes the relative orientation w.r.t. the robot’s global orientation. Arrows indicate a relative orientation of 0°, 90°, 180° and 270°. (c) Due to the periodic image boundary we can simulate a full rotation with the sliding window approach. The part of the image covered by the window represents the data that is processed at one time step. The size of the sliding window is given as the percentage of the original panoramic view.

For the task of self-localization, we want to find functions that encode the robot’s position on the *x*- and *z*-axis as slowly varying features and are invariant with respect to its orientation. As stated above, learned slow features strongly depend on the movement statistics of the mobile robot during the training run. In order to achieve orientation invariance, the orientation of the robot has to change on a faster timescale than its position. A constantly rotating robot with a fixed camera is inconvenient to drive, and a robot with a rotating camera is undesirable for mechanical stability and simplicity. As an alternative, we use an omnidirectional mirror which allows to easily add simulated rotational movement of the robot to manipulate movement statistics. Thus, the model is able to find orientation-invariant representations of its own position without having to rotate the camera or the robot physically. During the training phase we simulate a full rotation for every captured image. Since for panoramic images a lateral shift is equivalent to a rotation around the yaw axis we can simulate a full rotation by shifting a sliding window over the periodic panoramic views (see Fig 1 for an illustration). Throughout the experiments we use a window size equal to 100% of the image size so that each rotated view contains the whole image, incrementally shifted along the lateral direction. A systematic analysis of varying the window size will be given in a later section.

### Network architecture and training

As input image dimensionality is too high to learn slow features in a single step, we employ a hierarchical converging network. The network is made of several layers, each consisting of multiple SFA-nodes arranged on a regular grid. Each node performs a sequence of steps: linear SFA for dimensionality reduction, quadratic expansion of the reduced signals, and another SFA-step for slow feature extraction. The nodes in the lowest layer process patches of 10 × 10 gray-scale image pixels and are positioned every five pixels. In the lower layers the number of nodes and their dimensionality depends on the concrete setting, but dimensionality is chosen to be a maximum of 300 for numerical stability. The region of the input data visible to a node increases with every subsequent layer. The highest layer contains a single node, whose first (i.e., slowest) 8 outputs *g*_*j*_(*x*) we use for all experiments and which we call SFA-output units.

**Fig 2:**
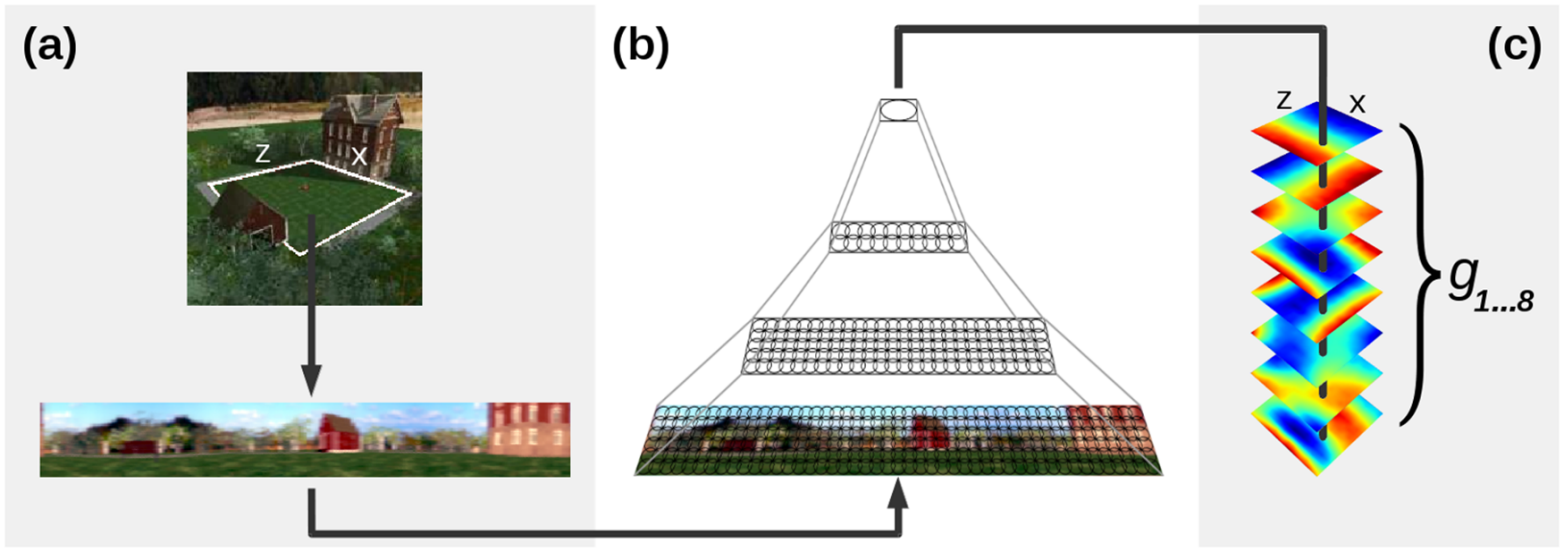
Model architecture. (a) The robot’s view associated with a certain position *r* = (*x*, *z*) is steadily captured and transformed to a panoramic view. (b) The view is processed by the four layer network. Numbers of nodes in each layer are given for the simulator (gray) and real world (black) experiments, respectively. Each node performs linear SFA for dimensionality reduction followed by SFA on the expanded outputs for slow feature extraction. (c) Eight slowest SFA-outputs *g*_1…8_ over all positions *r*. The color coded outputs, so-called spatial firing maps, ideally show characteristic gradients along the coordinate axes and look the same independent of the specific orientation. Thus SFA outputs *g*_1…8_ at position *r* are the orientation invariant encoding of location.

The layers are trained subsequently with all temporally ordered training images. A full rotation is simulated for every panoramic image by incrementally shifting it laterally by five pixels. For panoramic images a rotation on the spot around the yaw axis is equivalent to laterally shifting the image. Instead of training each node individually, a single node per layer is trained with stimuli from all node locations in its layer and replicated throughout the layer after training. This technique is similar to weight sharing in Neural Networks. Note that this design is chosen only for its computational efficiency and that network performance increases for individually learned nodes. After training the eight slowest SFA-outputs *g*_1…8_ are the orientation invariant encoding of the robot’s location and are computed instantaneously from a single image. An illustration of the model is given in Fig 2.

### Analysis of learned representations

How well does a learned output encode position, how much orientation dependency does it have? According to [7], the sensitivity of a SFA-output function *g*_*j*_, *j* = 1…8 to the spatial position ***r*** = (*x*, *z*) is characterized by its mean positional variance *η*_***r***_ over all orientations *φ*: *η*_***r***_ = 〈var_***r***_(*f*(***r***, *φ*))〉_*φ*_. Similarly, the sensitivity to the orientation *φ* is characterized by its mean orientation variance *η*_*φ*_ over all positions ***r***: *η*_*φ*_ = 〈var_*φ*_(*f*(***r***, *φ*))〉_***r***_. In the ideal case *η*_***r***_ = 1 and *η*_*φ*_ = 0, if a function only codes for the robot’s position on the *x*- and *z*-axis and is completely orientation invariant. The spatial information encoded by an output will be visualized by two dimensional spatial firing maps (see Fig 2c). They illustrate the unit’s output value color-coded for every position ***r*** = (*x*, *z*) for a fixed orientation, which is indicated by an arrow. A unit which codes for the position on a certain axis produces a map that shows a color gradient along this axis. If the SFA-units are perfectly orientation invariant these maps should look the same regardless of the specific orientation.

### Data and ground truth acquisition

#### Simulator

The images for the experiments were generated using a simple garden-like simulator environment (cf. Fig 1a and 1c). The virtual robot was placed on discrete positions forming a regular 30 × 30 grid. The images from every position were rendered and saved together with the corresponding coordinates. Trajectories for generating the training and test image sequences were artificially generated by traversing successive positions on the grid. We recorded 624 omnidirectional RGB images for the training set and 196 for the test set and transformed them to panoramic views with a resolution of 350 × 40 pixels. In the simulator environment we have perfect knowledge of the robot’s ground truth position which allows to easily asses localization accuracy.

#### Real world

The SFA-model processes omnidirectional images in order to facilitate learning of orientation invariant representations while LSD- and ORB-SLAM require a calibrated camera operating at a high framerate. Therefore two distinct cameras were used for image acquisition. The omnidirectional camera captures images with a framerate of 8 fps and is mounted above the marker box. For the SLAM methods we used a global shutter camera, equipped with a fisheye lens and operating at a framerate of 40 fps. Camera and lens are equal to the ones used by the authors of [19]. The camera was mounted on the front side of the robot heading orthogonal to the driving direction. This setup was chosen to enable wider baseline stereo correspondences and to enhance the robustness of the tracking during rotational movement. In case of a limited field of view, forward and rotational movement leads to correspondences with a small baseline between successive keyframes which increases the depth ambiguity and might cause a complete failure of the tracking system. Results with a forward facing cameras were systematically worse.

Images and ground truth coordinates are saved together with the current timestamp to enable the synchronization of image data and ground truth measurements. The offset from the cameras to the center of the marker box is measured manually and integrated into the ground truth computation. Exposure of both cameras was set automatically to account for changing lighting conditions during the recordings. Images of the perspective camera are captured in grayscale with a resolution 752 × 480 pixels. The undistorted images are cropped to 640 × 480 pixels. The omnidirectional images are unwarped to panoramic views with a resolution of 409 × 40 pixels and converted to grayscale. The image data is then normalized to zero mean and unit variance to gain robustness against global illumination changes. The rough terrain in the outdoor environment causes changes in the tilt angle of the robot. Thus image statistics from the same place with different physical orientations are not the same and our orientation invariance learning does not work anymore. Therefore we randomly shifted the center of every omnidirectional image by an offset from −5 to 5 pixels for the computation of the panoramic views. This way the resulting representations become invariant with respect to the tilt angle of the robot.

**Fig 3:**
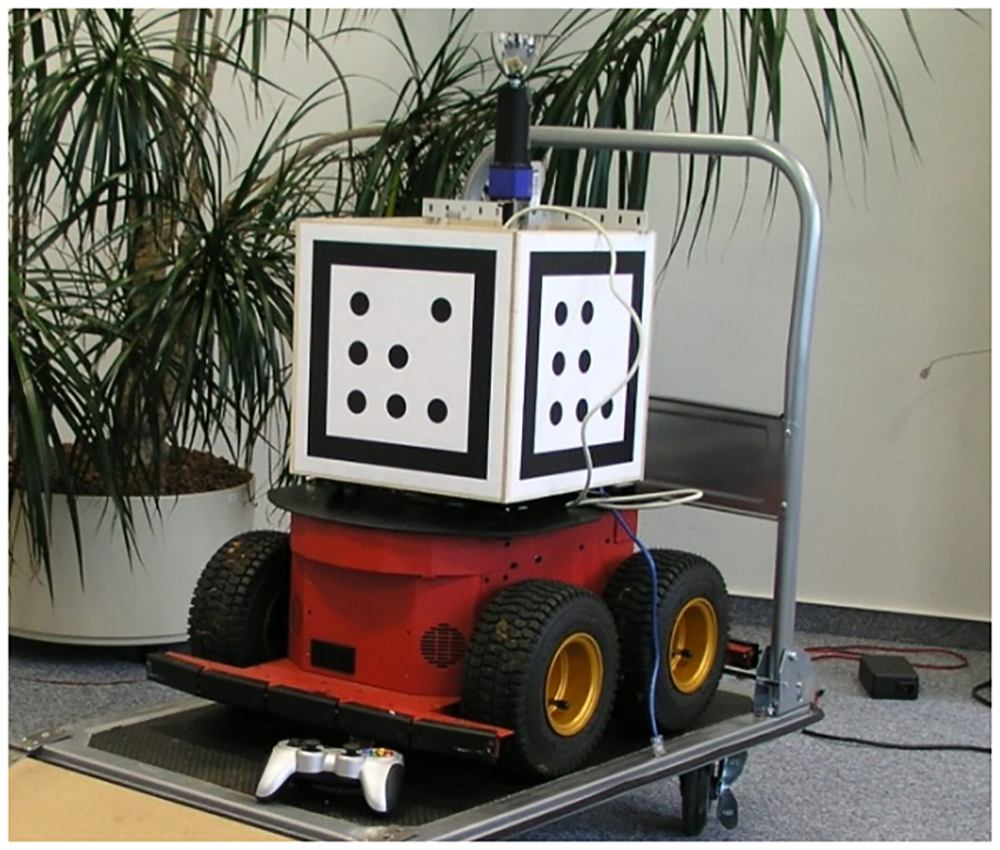
Pioneer 3AT equipped with omnidirectional vision system and marker-box.

To asses the localization performance in a metric way the true position of the robot has to be acquired independently through an external monitoring system. To keep ground truth acquisition flexible and robust we mounted a 30cm cube on the robot with optical, binary markers attached to its facets (Fig 3).

A stationary camera was installed to capture images of the whole area throughout the training- and test-runs. The robot’s 3d-pose was computed, based on the features of the detected markers, by solving the Perspective-n-Point problem. The Implementation is based on the OpenCV-library [22]. In an experimental setup with a high resolution camera the method provided a detection up to a distance of 18 meters with a mean deviation of 3.4cm, as verified by laser distance meter.

## Experimental results

The camera images and metric coordinates of the robot are steadily captured over the trajectories of the training- and test-runs. While the resulting SFA-units may already be sufficient for performing a navigation task, we want to have a metric measure to assess localization performance. Therefore we compute a regression function from the SFA-outputs of the training run to the metric ground truth positions and subsequently apply it to SFA-outputs of the test run. This allows to quantify and visualize the encoded spatial information of the SFA-outputs in a metric way.

### Simulated environment

The model was first applied in a virtual reality simulator to validate the model under entirely controllable settings and to present an analysis of the spatial encoding resulting from optimal conditions. The network architecture, defined by the number of layers, the arrangement of the receptive fields (RF) and their dimensionality, is given in Table 1.

**Table 1:** Network parameters for the simulator experiment.

Layer	Number of RFs (w×h)	RF size (w×h)	Stride (w×h)	Input dim	Output dim
1	69 × 7	10 × 10	5 × 5	300	14
2	22 × 3	6 × 3	3 × 2	252	16
3	10 × 1	4 × 3	2 × 1	192	16
4	1 × 1	1 × 1	1 × 1	160	8

Number of Receptive Fields (RF) per layer, RF size and stride are given for every layer of the SFA network.

#### Results

**Fig 4:**
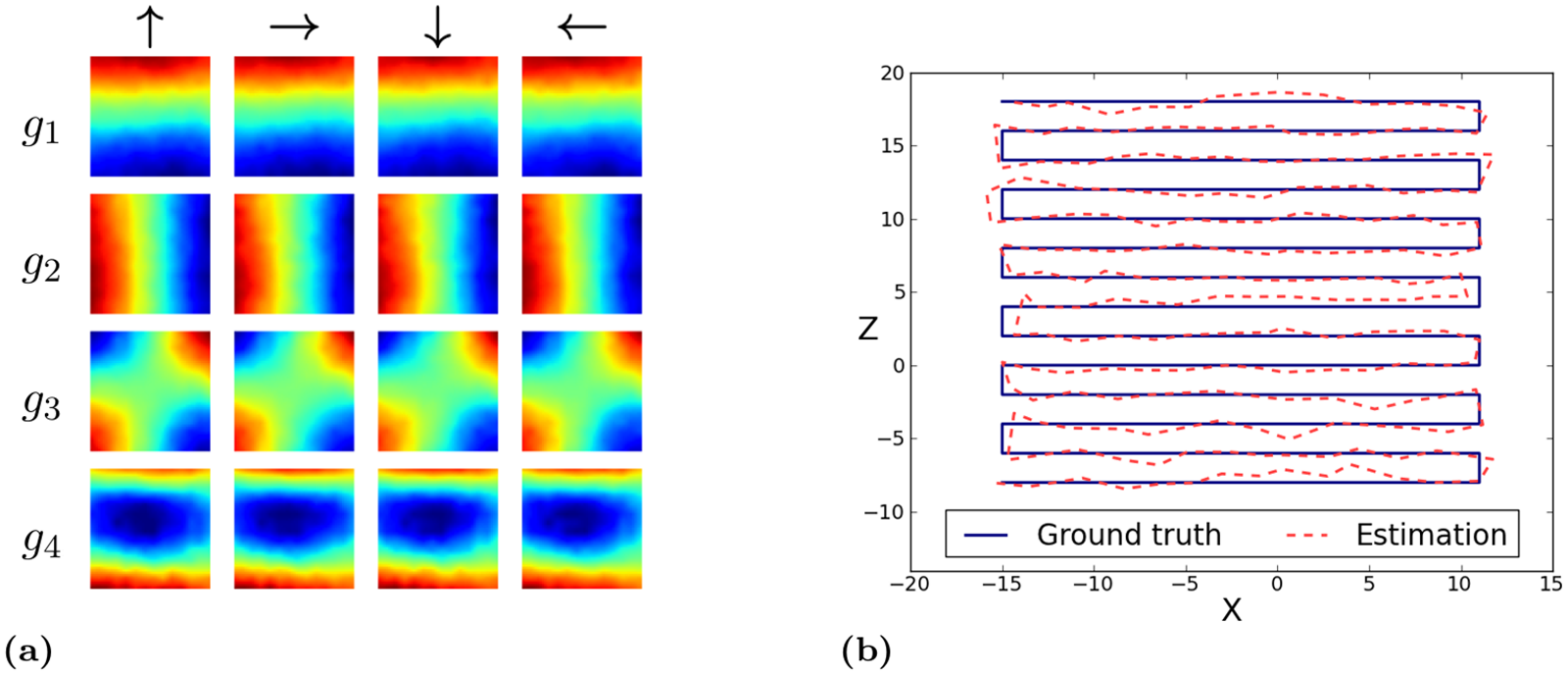
Simulated environment. (a) Spatial firing maps of the four slowest SFA-outputs *g*_1…4_ for relative orientations 0°, 90°, 180° and 270°. Obviously the first and second outputs are spatially orthogonal, coding for *z*- and *x*-position, respectively. Output values are monotonically increasing from north to south and east to west. The third unit is a mixture of the first two units and unit four is a higher oscillating representation of the first unit. (b) Ground truth and estimated coordinates computed by the regression. Estimations are averaged over the windows of the simulated rotation for one location.

All resulting SFA-units have a high spatial structure and are almost completely orientation invariant as their outputs for the training views have a mean positional variance *η*_*r*_ ≈ 1 and the mean orientation variance *η*_*φ*_ ranges from 0.00 (*g*_1_) to 0.17 (*g*_8_). This is also reflected by the spatial firing maps in Fig 4a which show an obvious encoding for the position on the coordinate axes and look nearly identical under different orientations. These results are very similar to the theoretically predicted optimal SFA solutions given in [7].

Since here in the simulator, the views of the training- and test-run are identical for the same location we only use the test data for the regression analysis. Random 50/50 splits are used to train the regression and evaluate the coordinate prediction. Repeating it 100 times results in an overall mean absolute error (*MAE*) for the *x*- and *z*-coordinate estimation of 1.83% and 1.68%, relative to the coordinate range of the test run (Fig 4b).

### The impact of the window size

**Fig 5:**
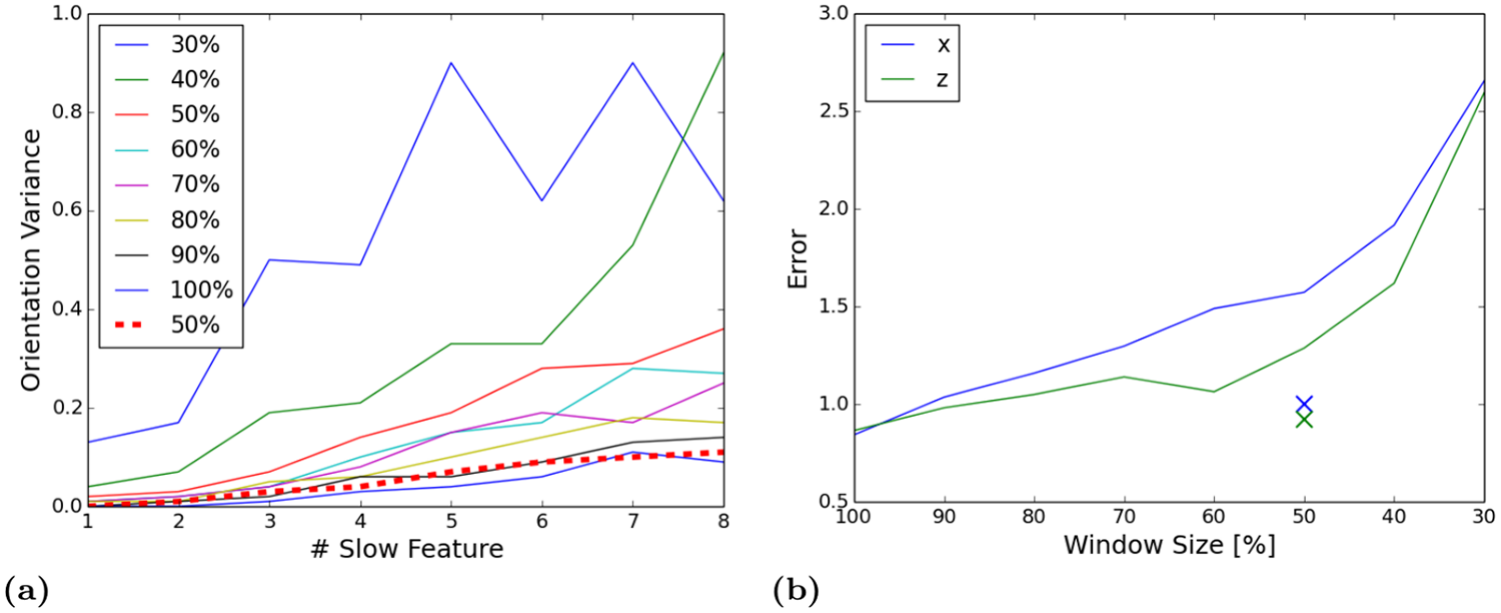
Effect of different window sizes. (a) The encoded orientation variance of the eight slowest features for different window sizes. Variance of the modified network with a 50% window is shown by the red dotted line. (b) Localization error of networks trained with different window sizes. Error of the modified network is indicated by crosses. Performance of the modified network ranges between the original network trained with 90% and 100% windows.

Learning location specific and orientation invariant functions with the SFA-model requires that the orientation of the robot changes on a faster timescale than its translation, since the spatial encoding of the SFA-Model depends on the movement statistics during training. To change the perceived image statistics a complete rotation is simulated for every image by laterally sliding a window over the periodic panoramic views. For panoramic images a lateral shift is equivalent to a rotation of the image sensor on the spot around its yaw axis. In the experiments we used a window size of 100% which means that the whole image is processed by the model but incrementally shifted in every step of the simulated rotation. Learning with smaller windows would decrease the computational complexity. However, experiments with different window sizes show that the orientation variance, and hence the localization error, is increasing with smaller windows. This effect is illustrated in Fig 5a and 5b.

We conjecture that the complexity of the learning problem is getting too high if the window size is reduced. For an optimal performance the output of the functions should be nearly constant for the input perceived during a simulated rotation and vary after a change in position. During a simulated rotation with a 100% window the output SFA-node receives statistics from the whole image with a lateral shift for every step. With decreasing window sizes, however, the input statistics perceived by the output node vary increasingly which requires learning more complex functions. To increase the function space the expansion of the input data was changed from quadratic to cubic. Furthermore, the learning problem was simplified by training individual SFA-nodes per receptive field instead of sharing the weights across one layer. Experiments with these modifications and a window size of 50% resulted in a considerable improvement for the simulated environment (see Fig 5a and 5b). For the non-static and noisy outdoor data-sets the encoding of the location did not improve with the complex model and 50% windows and it is hard to determine which information is extracted from the expanded high dimensional input. Depending on the requirements a tradeoff between mapping quality and computation time has to be made. Thus, we use a window size of 100% throughout the experiments.

### Real world experiments

To investigate the localization capabilities in a realistic setting we applied the SFA-model and SLAM-methods in open field indoor and outdoor scenarios with image data captured from trajectories driven by a mobile robot. The robot was moved with a wireless joystick during the training- and test-runs at a maximum velocity of 20 cm/s. Localization accuracy was evaluated for the test run only. The SFA-model requires an offline training phase to learn the spatial representation of the environment. SLAM methods, on the other hand, perform mapping and pose estimation incrementally and online, which is why the localization accuracy can be evaluated on the test run directly. To make a fair comparison we also provided the SLAM-methods with image data from the training and test run and measured the performance on the test run. We used the the default configuration given by the authors and executed the SLAM-methods in mapping mode for all experiments to allow for map updates and pose correction in the subsequent test run. Their localization accuracy is evaluated over five runs since the results are non-deterministic due to the parallel execution of the mapping and tracking threads.

The parameters of the SFA-network used in the real world experiments are given in Table 2. To evaluate the localization accuracy the estimated trajectories are aligned to the ground truth trajectories by finding the rotation and translation between the two 3d-point sets which minimizes the mean squared error as described in [23]. As the absolute scale can not be recovered from a single camera we perform the fitting over a predefined scale range.

**Table 2:** Network parameters for the real world experiments.

Layer	Number of RFs (w×h)	RF size (w×h)	Stride (w×h)	Input dim	Output dim
1	101 × 7	9 × 10	4 × 5	90	12
2	49 × 2	5 × 5	2 × 2	300	12
3	23 × 1	5 × 2	2 × 1	120	12
4	1 × 1	1 × 1	1 × 1	276	8

Number of Receptive Fields (RF) per layer, RF size and stride are given for every layer of the SFA network.

#### Indoor environment

The datasets for the experiments were recorded in an indoor environment covering an area of about 4 × 4 meters. Two experiments with different movement characteristics have been performed since it influences the mapping results of the methods in different ways. The training trajectory for both experiments evenly samples the area with crossings along the coordinate axis resulting in a grid-like pattern. In the first experiment turn maneuvers were executed with a large curve radius while the robot was turned at the spot in the second experiment. Turning on the spot promotes the spatial encoding of the SFA-model because it naturally leads to a larger amount of overlap between different parts of the trajectory for a similar track length. Crossing points in the trajectory ensure that image data from the same place at different points in time are presented to the SFA-model which improves spatial encoding. Pure rotational movement during the mapping phase is problematic for the SLAM-methods since the camera motion and depth estimation requires a certain amount of translation between successive frames. Larger curve radii are thus necessary to achieve a good ratio of rotational and translational movement. In principle this turn characteristic does not pose a problem to the SFA-model but might decrease the quality of the spatial representation since the overlap of the trajectory is quite low compared to trajectories of the same length where the robot turns on the spot.

#### Experiment I

**Fig 6:**
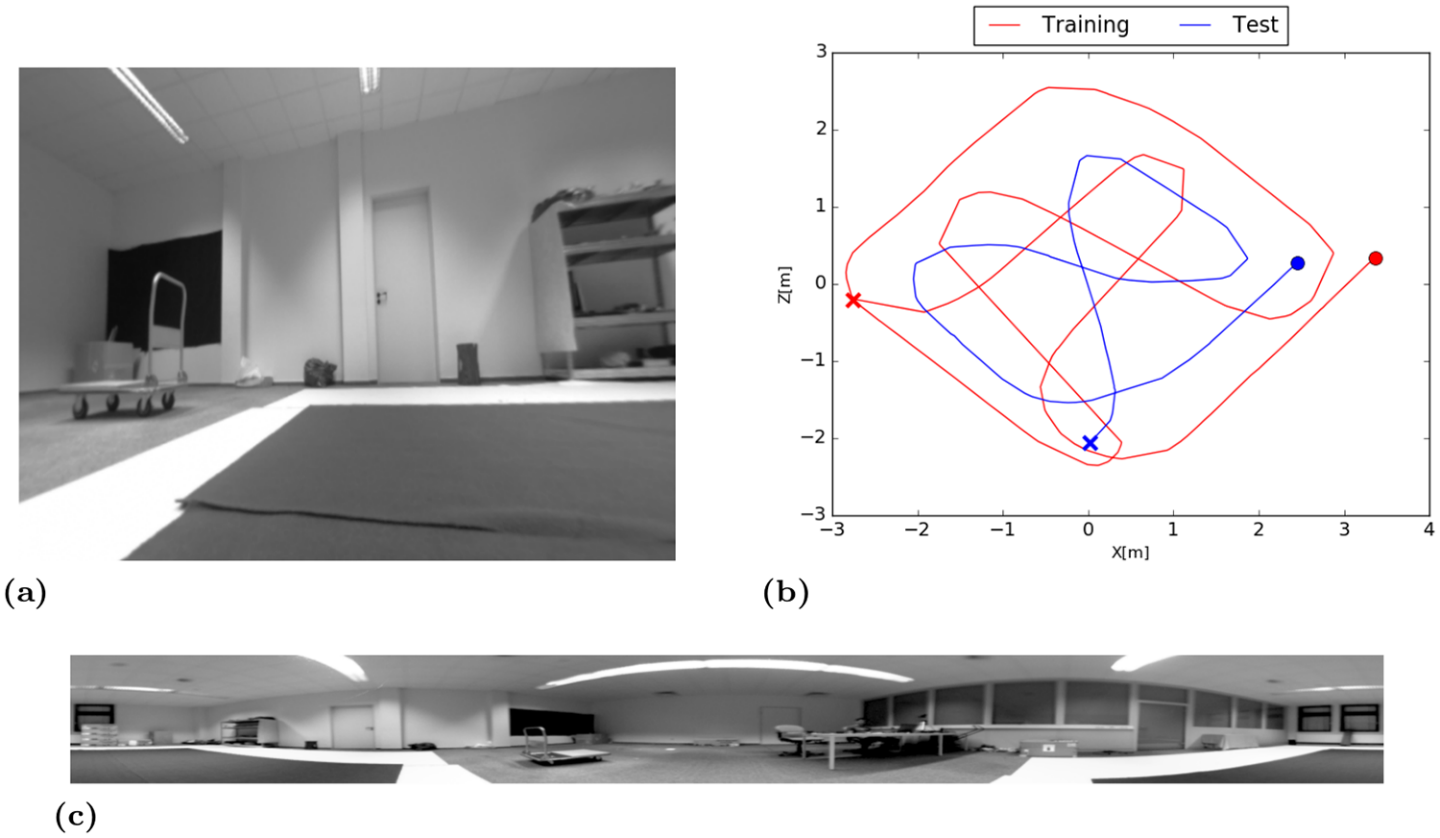
Indoor environment. (a) Undistorted image from the perspective camera mounted on the side of the robot. (b) Trajectory of the training- and test-run. Start and end points are marked by a cross and a circle, respectively. (c) Panoramic image captured by the omnidirectional camera.

The trajectory follows a grid-like structure that evenly covers the training area. Turn maneuvers were performed with a large curve radius. As stated above this ensures a proper ratio of rotational and translational movement required by SLAM-methods during the mapping phase while this is not optimal for the SFA-model. The trajectory of the training- and test-runs are given in Fig 6a. Example images from both cameras are illustrated in Fig 6b and 6c.

#### Results

**Fig 7:**
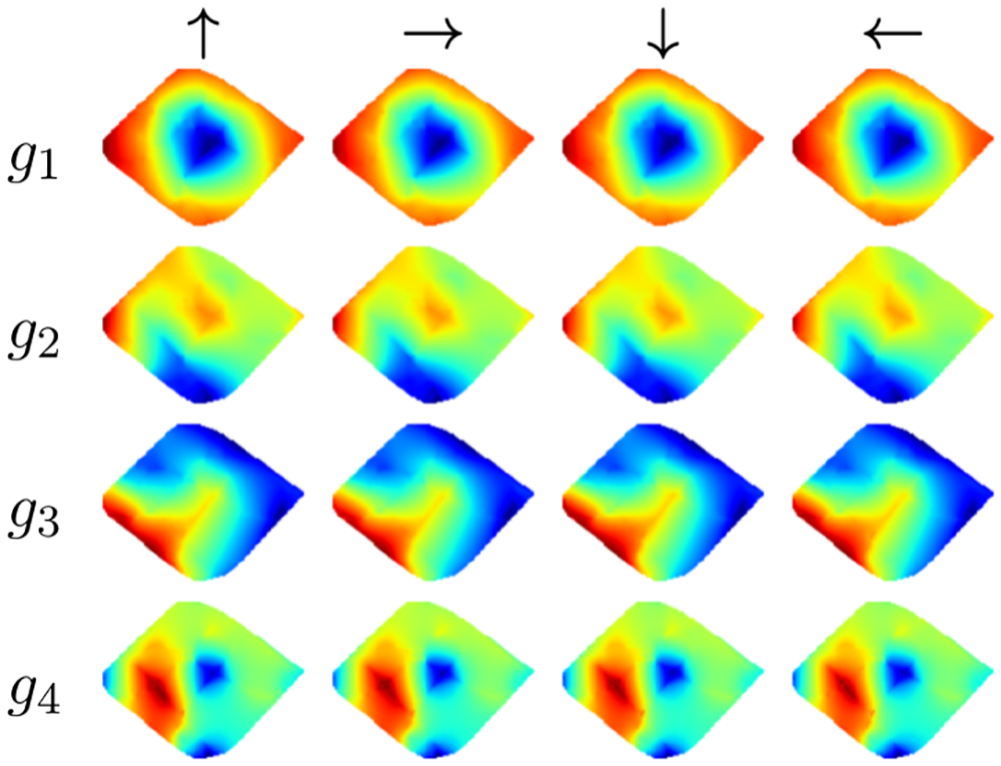
Spatial firing maps of the first four SFA-units. First unit seems to be encoding the distance to the borders of the area. Units two and three suggest encoding of the *x*- and *z*-position while the gradients along the coordinate axis are not as clear as in the simulator experiments. All units are highly orientation invariant.

The mean positional variance of the resulting SFA-units *η*_*r*_ is ≈ 1 and the mean orientation variance *η*_*φ*_ is ≈ 0. SFA-units thus have a high spatial structure and are almost completely orientation invariant. The spatial firing maps of the four slowest SFA-units shown in Fig 7 do not show an obvious encoding of the position with clear gradients along the coordinate axis as in the simulator experiment.

The first unit seems to be coding for the distance to the borders while units two and three suggest coding for the *x*- and *z*-coordinate. The mean Euclidean distance is 0.21m. The best localization performance is achieved with LSD-Slam with a median localization error of 0.19m when the train- and test-images are used and an error of 0.12m when using the test-images alone. The accuracy is quite constant over the runs except for the fifth run on the test data.

**Fig 8:**
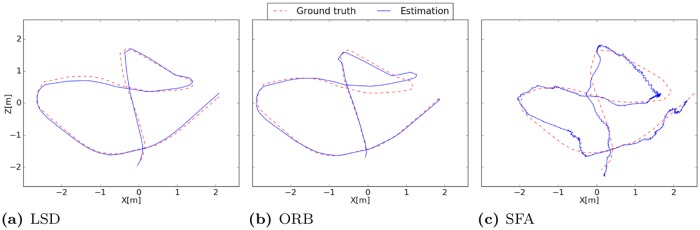
Estimated trajectories of the best runs. (a) The trajectory estimated by LSD-Slam clearly follows the ground truth with small deviations. (b) Deviations in the trajectory produced by ORB-Slam start to get greater after the left turn where the curve radius of the camera is quite small. (c) Since the SFA-localization is absolute and no pose filtering is performed the trajectory is in general more noisy. The accuracy decreases near the borders.

The accuracy of ORB-Slam amounts to a median error of 0.45m on the training- and test-images while the interquartile range of the five runs is quite high with 0.27m. On the test-images alone the variance of the errors is lower and the median error amounts to 0.23m. The performance of ORB-Slam probably suffers from the low textured indoor environment which is disadvantageous for the amount and distribution of robust visual features. Surprisingly the performance of the SLAM-methods is worse when images from the training run are used for the experiment. We expected that mapping quality would improve through the additional information from the training run. Instead the constructed pose-graphs often got corrupted due to tracking failures. The results are presented in detail in Table 3. The resulting trajectories of the best runs of the different methods are illustrated in Fig 8.

**Table 3:** Localization accuracies for indoor experiment I.

	Train- and Test-Run						Test-Run					
	1	2	3	4	5	Median	1	2	3	4	5	Median
**ORB**	0.22	0.45	0.57	0.49	0.12	**0.45**	0.25	0.25	0.16	0.18	0.23	**0.23**
**LSD**	0.17	0.30	0.18	0.15	0.38	**0.18**	0.11	0.11	0.13	0.12	0.40	**0.12**
**SFA**	**0.21**											

Localization errors are given in meters as the mean Euclidean distance from all ground truth measurements. The performance of LSD- and ORB-Slam is measured over five runs since the results are not deterministic due to the parallel execution of the tracking and mapping threads. The SFA-localization requires an offline training phase and is deterministic thus only one measurement of the error is given.

#### Experiment II

**Fig 9:**
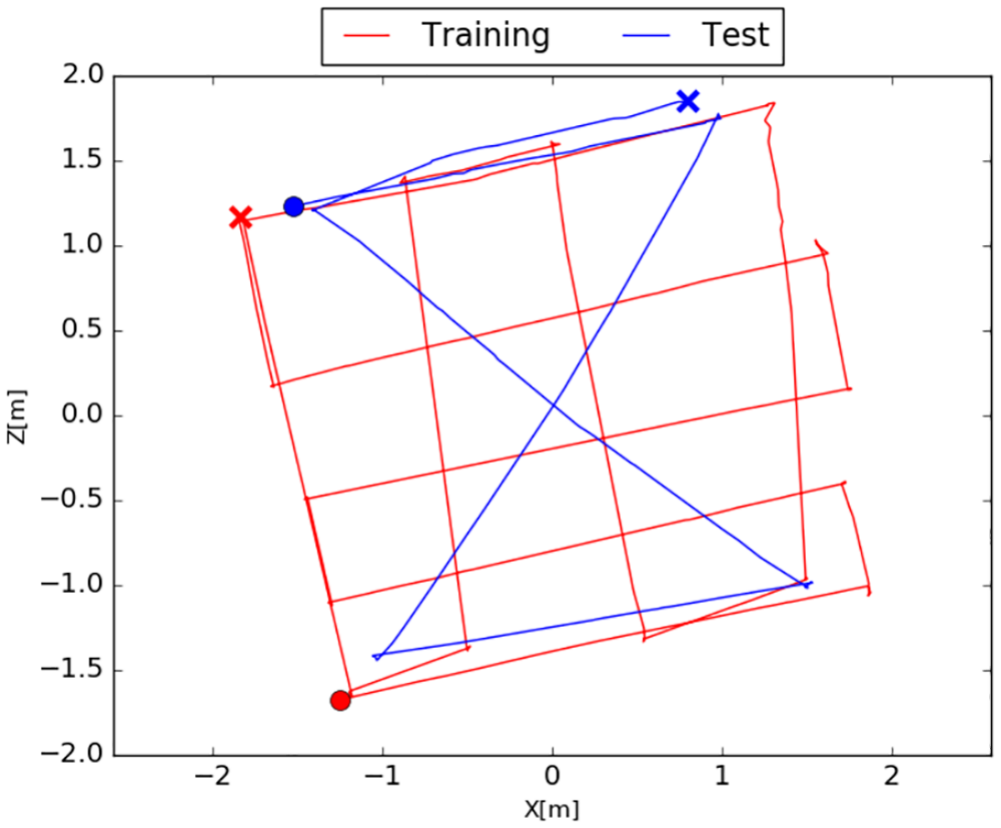
Training- and test-trajectory of the second experiment which is more favorable for the SFA-localization because of more crossing points and a denser sampling of the area. Trajectories are challenging for the SLAM-methods because of the high amount of rotational movement. Start and end points are marked by a cross and a circle, respectively.

The second experiment was conducted with a different movement strategy (see Fig 9). Turning maneuvers were performed on the spot resulting in a denser sampling of the area and larger overlaps in the trajectory which is beneficial for the SFA-localization. Monocular SLAM-methods on the other hand have problems with pure rotational movement since it is not possible to triangulate features without a sufficiently large baseline so that they easily lose tracking. The Movement strategy for SFA is only relevant in the training phase while it works for every trajectory in testing.

#### Results

**Fig 10:**
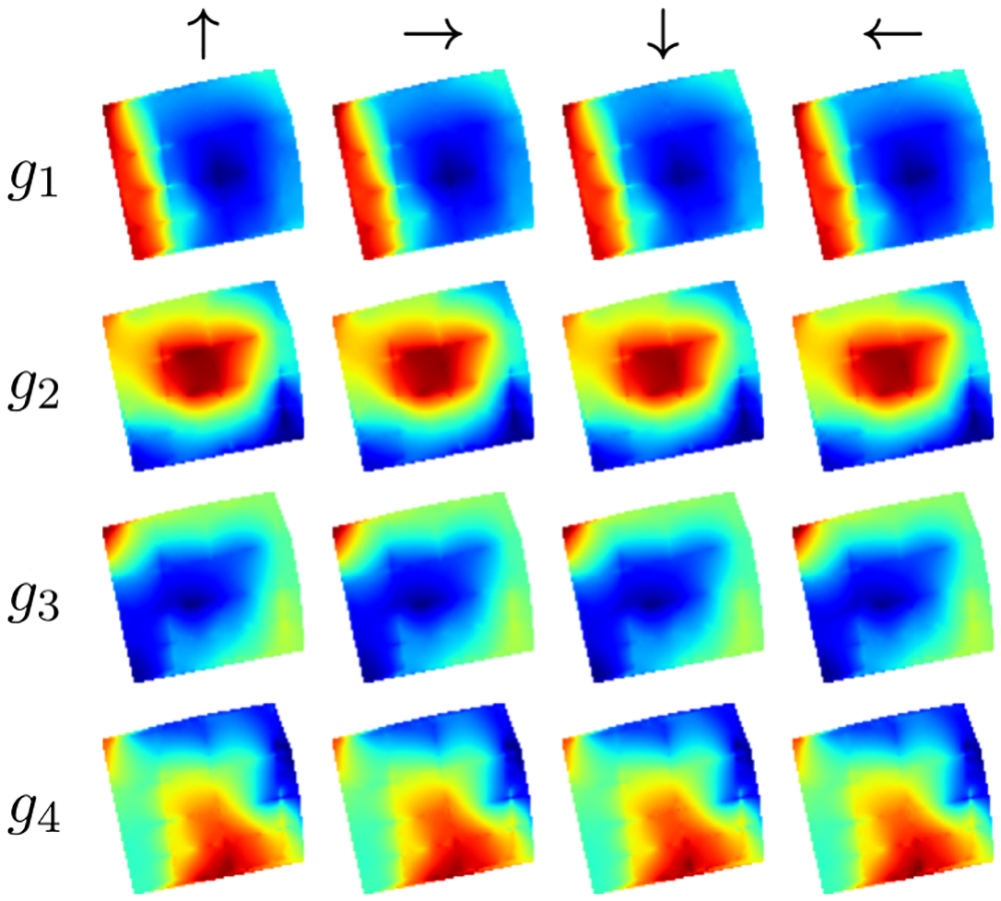
Spatial firing maps of the four slowest SFA-units. First two units seem to be encoding the distance to the borders but also show a gradient along the coordinate axis.

**Fig 11:**
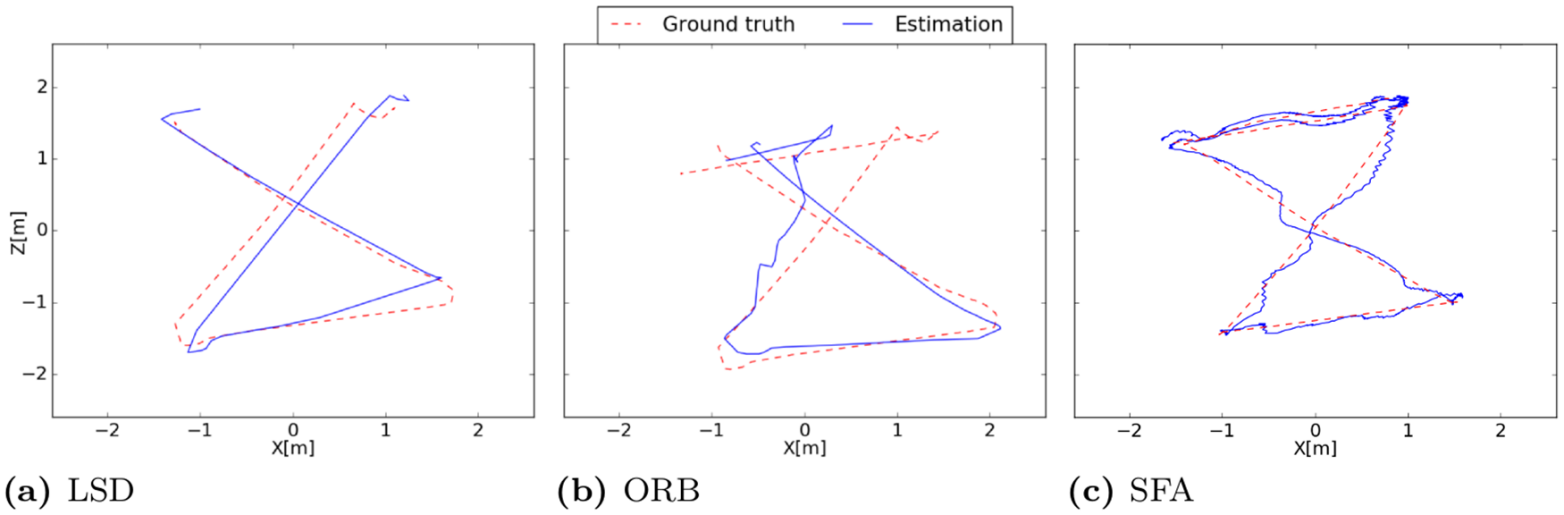
Estimated trajectories of the best runs. (a) LSD-Slam is not able to re-localize during the first seconds so that there are no pose estimates available. Map quality decreases after the third turn. (b) ORB-Slam is instantaneously able to re-localize in the map and the pose estimates are close to the ground truth until the third turn. (c) The estimated trajectory of the SFA-model clearly follows the ground truth.

As in the first experiment SFA-units have a high spatial structure while being invariant with respect to the orientation of the robot with a mean positional variance *η*_*r*_ of ≈ 1 and a mean orientation variance *η*_*φ*_ of ≈ 0. The spatial firing maps presented in Fig 10 again seem to be encoding the distance to the center mixed with positional encoding which can be seen by a gradient along the coordinate axis. The best localization performance is achieved by the SFA-model with a mean localization error of 0.13m. Both SLAM-methods fail completely on the test trajectory alone. When using the training and test data tracking failures during the test run are retained by a re-localization. The median localization errors of ORB- and LSD-Slam amount to 0.78m and 0.44m respectively. The results are presented in detail in Table 4. The resulting trajectories of the best runs of the different methods are illustrated in Fig 11.

**Table 4:** Localization accuracies for indoor experiment II.

	Train- and Test-Run						Test-Run					
	1	2	3	4	5	Median	1	2	3	4	5	Median
**ORB**	0.41	1.23	1.01	0.78	0.46	**0.78**	1.34	1.34	1.34	1.34	1.34	**1.34**
**LSD**	0.27	0.58	0.75	0.22	0.44	**0.44**	1.10	1.33	1.08	1.08	1.32	**1.10**
**SFA**	**0.13**											

Localization errors are given in meters as the mean Euclidean distance from all ground truth measurements. The performance of LSD- and ORB-Slam is measured over five runs since the results are not deterministic due to the parallel execution of the tracking and mapping threads. The SFA-localization requires an offline training phase and is deterministic thus only one measurement of the error is given.

#### Outdoor environment

**Fig 12:**
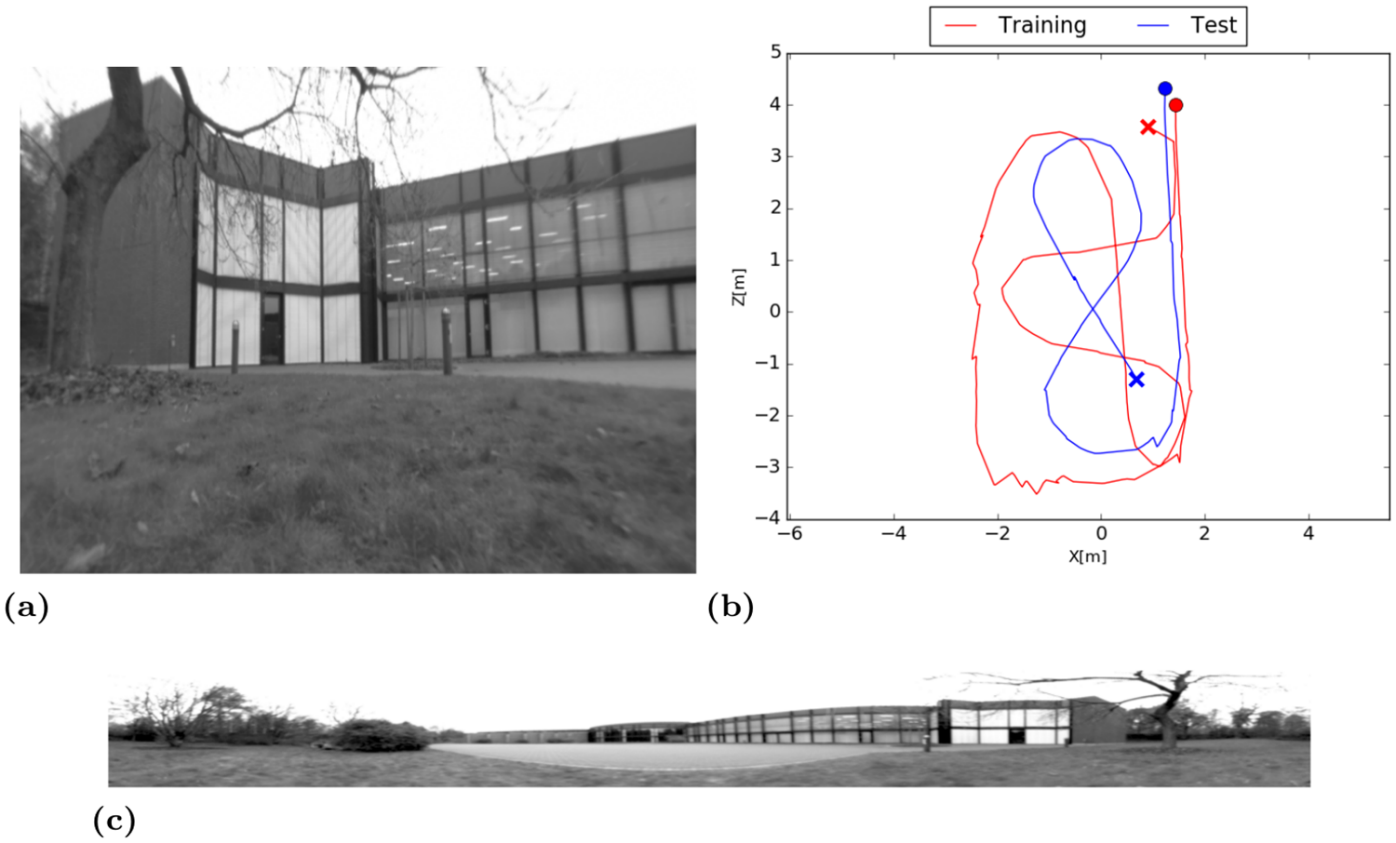
Outdoor environment. (a) Undistorted image from the perspective camera mounted on the side of the robot. (b) Trajectory of the training- and test-run. Start and end points are marked by a cross and a circle, respectively. (c) Panoramic image captured by the omnidirectional camera.

Outdoor experiments were done within an area of approximately 5 × 7 meters on rather uneven ground covered by grass. Recordings were done in the late afternoon with modest changes in lighting conditions. The trajectory of the training- and test-run are given in Fig 12b. Example images from both cameras are illustrated in Fig 12a and 12c.

#### Results

**Fig 13:**
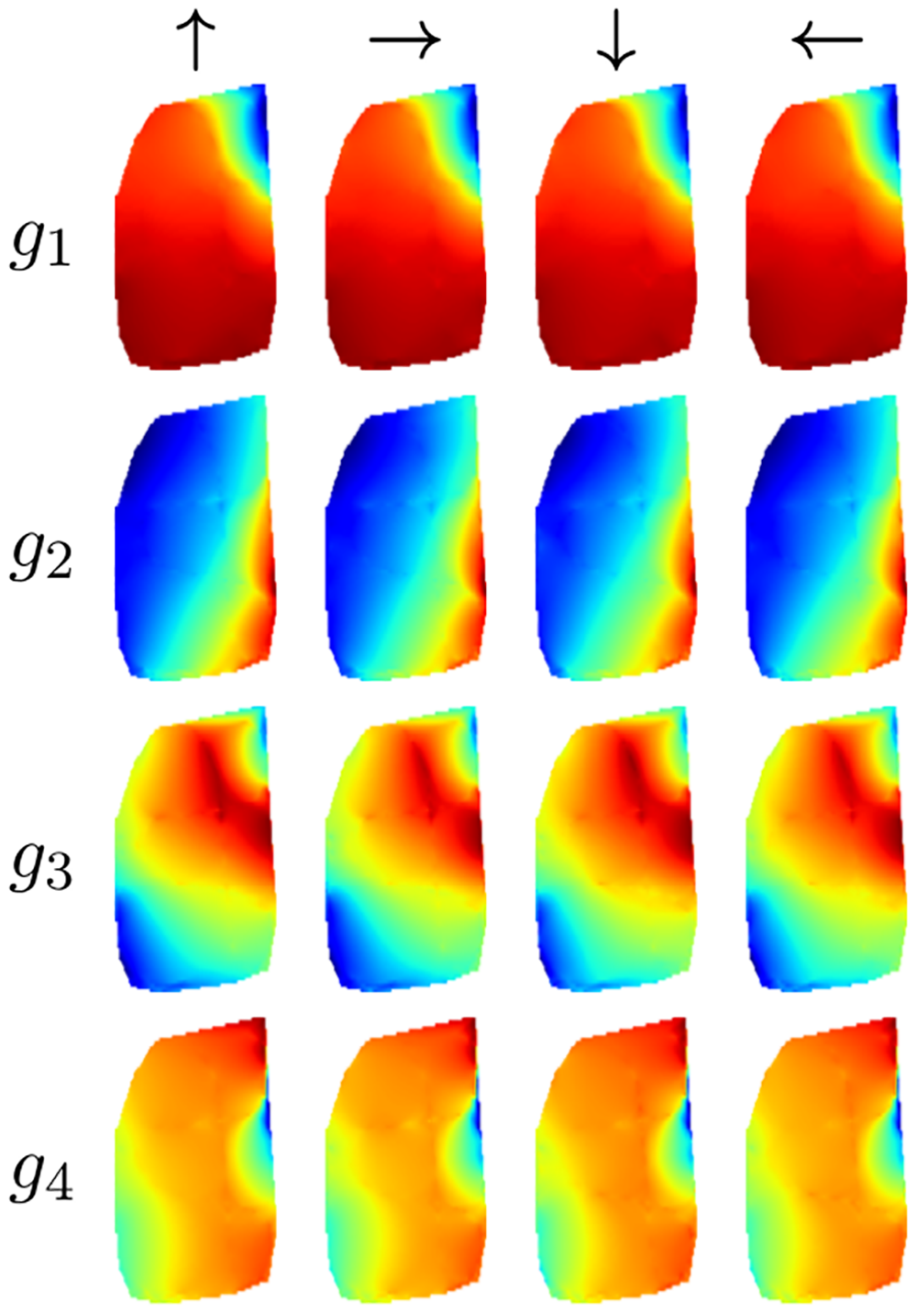
Spatial firing maps of the four slowest SFA-units. First two SFA-units show spatial encoding while directions in the data with least temporal variation are slightly rotated with respect to the coordinate axis. This can be the case if temporal variation of the *x*- and *z*-coordinate is nearly equal. Units three and four are higher modes of the first two units.

The resulting SFA-units show a clear spatial coding and are orientation invariant. Spatial firing maps illustrated in Fig 13 show a slightly rotated gradient along the coordinate axis.

**Fig 14:**
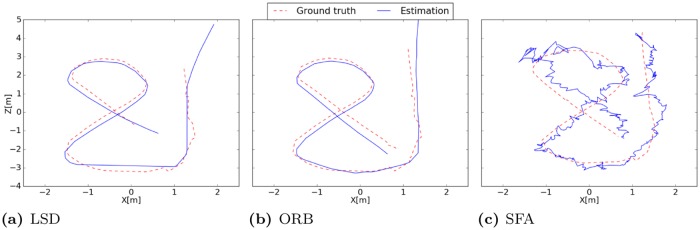
Estimated trajectories of the best runs. (a) Estimated trajectory of LSD-Slam clearly follows the ground truth while the scale is estimated incorrectly in the beginning of the trajectory. (b) ORB-Slam also has problems with scale estimation in the beginning. The best performance is achieved on the test data alone where only one loop closure occurs so that the estimation starts to drift over time. (c) SFA estimates have a higher variance which can be explained by the uneven ground. The instantaneous and absolute position estimates result in the best performance for this experiment.

Due to the uneven ground and the difficult lighting conditions the dataset is challenging for all methods. Both SLAM methods have problems with scale estimation in the first part of the trajectory leading to larger errors in the localization. Even though the trajectory of the SFA-model exhibits larger variance in local estimates the performance is best on this data set with a mean localization error of 0.33m. Due to the instantaneous and absolute localization the model is not affected by drift over time. ORB-Slam achieves a median accuracy of 0.35m on the test data alone followed by LSD-Slam with a median accuracy of 0.44m when the training and test data is used. The results are presented in detail in Table 5. The resulting trajectories of the best runs of the different methods are illustrated in Fig 14.

**Table 5:** Localization accuracies for the outdoor experiment.

	Train- and Test-Run						Test-Run					
	1	2	3	4	5	Median	1	2	3	4	5	Median
**ORB**	0.99	0.52	1.86	0.61	1.16	**0.99**	0.35	0.35	0.71	0.63	0.34	**0.35**
**LSD**	0.47	0.60	0.69	0.53	0.44	**0.53**	1.50	1.52	1.49	1.51	1.55	**1.51**
**SFA**	**0.33**											

Localization errors are given in meters as the mean Euclidean distance from all ground truth measurements. The performance of LSD- and ORB-Slam is measured over five runs since the results are not deterministic due to the parallel execution of the tracking and mapping threads. The SFA-localization requires an offline training phase and is deterministic thus only one measurement of the error is given. In this experiment the instantaneous and absolute position estimates from the SFA-model result in the best performance.

## Conclusion

We presented a biologically motivated model for visual self-localization based on the principle of slowness learning. The model extracts spatial representations of the environment by directly processing raw high-dimensional image data in a hierarchical SFA-network employing a single unsupervised learning rule. The use of an omnidirectional vision system allows to learn orientation invariant representations of the location by modifying the perceived image statistics with additional simulated rotation. The resulting functions encode the position of the camera as slowly varying features while at the same time being invariant to its orientation.

We demonstrated the feasibility of the approach in a simulated environment and compared its performance to state of the art visual SLAM methods in real world indoor and outdoor experiments. Although the presented SFA-model is rather straightforward, in the sense that it applies the same unsupervised learning rule in a hierarchical network directly to the visual input from an uncalibrated image sensor, its localization performance is competitive to the more complex and modular SLAM systems and can even surpass them for certain trajectories. In contrast to the SLAM methods the SFA-model requires an offline learning phase with an even sampling of the area. After the training phase localization is instantaneous and absolute which obviates dealing with drift over time and re-localization. The training trajectory has to include a certain amount of crossings to support spatial coding which renders SFA inappropriate for localization along one dimensional routes like road tracks. Potential application domains could be service robotics which require localization in open field scenarios. Since the spatial representations are directly learned from image data they are more susceptible to appearance changes in the environment than a feature based method. In future work we will investigate learning strategies and feature representations that improve robustness of the representations. The current SFA-model only uses visual input to localize while preliminary experiments show a substantial gain in accuracy when the SFA-estimates are fused with wheel odometry in a probabilistic filter.
